# Microplastics Contamination in Nonalcoholic Beverages from the Italian Market

**DOI:** 10.3390/ijerph20054122

**Published:** 2023-02-25

**Authors:** Arianna Crosta, Marco Parolini, Beatrice De Felice

**Affiliations:** Department of Environmental Science and Policy, University of Milan, via Celoria 26, I-20133 Milan, Italy

**Keywords:** beverages, human ingestion, microplastics, soft drinks

## Abstract

A growing number of studies have confirmed that microplastics (MPs) contamination represents a worrisome issue of global concern. MPs have been detected in the atmosphere, in aquatic and terrestrial ecosystems, as well as in the biota. Moreover, MPs have been recently detected in some food products and in drinking water. However, only limited information is currently available for beverages, although they are largely consumed by humans and might contribute to the ingestion of MPs. Thus, estimating the contamination in beverages represents a crucial step in assessing human MP ingestion. The aim of the present study was to explore the presence of MPs in nonalcoholic beverages, namely soft drinks and cold tea, of different brands purchased in supermarkets and to estimate the contribution of beverage consumption to MP ingestion by humans. The results of the present study confirmed the presence of MPs, mainly fibers, in most of the analyzed beverages, with a mean (± SEM) number of 9.19 ± 1.84 MPs/L. In detail, the number of MPs detected in soft drinks and cold tea was 9.94 ± 0.33 MPs/L and 7.11 ± 2.62 MPs/L, respectively. Our findings confirmed that beverage consumption can be considered one of the main pathways for MP ingestion by humans.

## 1. Introduction

Plastic pollution emerged as one of the main environmental issues at the global level. The increase in the demand, production, and use of plastics, together with the mismanagement and disposal of plastic waste at its end-of-life, have resulted in a massive contamination of both aquatic and terrestrial ecosystems worldwide [1]. In the environment, plastics can experience breakage, fragmentation, and degradation due to weathering activity mediated by chemical, physical, and biological processes, leading to the formation of small-sized items [2]. Among these, microplastics (MPs, i.e., plastic items <5 mm in size) represent one of the main environmental concerns at global scale because of their presence, distribution, and potential hazard to living organisms and ecosystems [3]. Several studies have demonstrated that MPs of different shape, size, color, and polymeric composition can be easily ingested and accumulated in a wide array of aquatic and terrestrial organisms [1,4]. Recent studies have also highlighted the presence of different types of MPs in humans. For instance, 21 different types of MPs (20–500 μm in size), whereby items made of polyurethane (PUR), polyester (PL), and chlorinated polyethylene (PE-C) dominated the fingerprint, have been detected as ubiquitous in human sputum [5]. Spherical or irregular polypropylene (PP) MPs (5–10 μm in size) have been detected in the human placenta [6], while the presence of polyethylene terephthalate (PET), polyethylene (PE), and polymers of styrene were detected in human blood at a total concentration of 1.6 µg/mL [7], demonstrating the distribution of MPs in different body districts. In addition, PP and PET fragments and films (50–500 µm in size) have been detected in human stool, confirming their transit through the human digestive system and egestion [8]. Humans can ingest MPs via different pathways, including inhalation, ingestion through hand-to-mouth contact, and the diet [9]. The main origin of MPs in humans is accountable to the intake of contaminated food and drinking water [10]. As MPs are efficiently ingested by marine and terrestrial organisms consumed as food, such as fish, mussels, crabs, chicken, and edible plants, they finally enter human bodies through the food chain [11]. Moreover, food and drinking water are commonly conserved in plastic packages, which may unintentionally release MPs and contaminate the product during production, transportation, and the packaging process [12]. Lastly, MPs can enter food and drinking water during processing, storage, transportation, and packaging processes [13], as well as through the deposition of aerial contamination in production areas. Contamination of MPs in food and drinking water has raised a topic of global concern, and a series of studies have investigated the presence of MPs in diverse food products [14,15], tap and drinking waters [13,16], as well as mineral waters [17,18]. Although water is the main ingredient in beverages and might be a major source of MPs [10], a limited number of studies have focused attention on this topic. Beverages include a wide range of alcoholic (i.e., beers, wines and spirits) and nonalcoholic (i.e., tea, coffee, milk, soft drinks, energy drinks, carbonated, and noncarbonated sweetened drinks) products. In 2021, the global consumption of nonalcoholic beverages in Europe amounted to 123,125.5 million liters, corresponding to 235.4 L pro capita [19]. Soft drinks made a remarkable contribution to beverage consumption (ca. 40%), with 48,288.4 million liters consumed, corresponding to 92.4 L pro capita [19]. It has been estimated that 9% of people aged 15 and over in the EU drink soft drinks daily, while 6% drink them 4–6 times a week, and 19% drink them 1–3 times a week. The highest share was recorded among young people aged 15 to 24 (14%), while the lowest shares (ca. 5%) were recorded for people aged 65 to 74 and >75 [20]. Among the EU Member States, the share of people reporting a consumption of soft drinks at least once a day was highest in Belgium (20%), followed by Malta, Germany, Hungary, Poland, and Bulgaria (ca. 12% for all the countries; Eurostat, 2019). Thus, considering the high consumption of beverages, some studies have investigated the presence of MPs in different products. To date, only a few studies have investigated the presence of MPs in beverages. MPs have been detected in 24 German-branded beers [21], whose contamination was higher compared with beers from Mexico [10]. MPs have also been found in milk samples from five international and three national brands of Mexico, with an average concentration of 6.5 ± 2.3 MPs/L [22]. In addition, MPs have also been detected in soft drinks, energy drinks, and cold tea purchased in Mexico, where the average contamination accounted for 40 ± 24.53 MPs/L, 14 ± 5.79 MPs/L, and 11 ± 5.26 MPs/L, respectively [10]. Thus, the aim of the present study was to investigate the contamination of MPs in two groups of nonalcoholic beverages, namely soft drinks and cold tea, of different brands purchased in Italian markets to expand the limited knowledge on this topic and estimate the contribution of beverage consumption to MP ingestion by humans. Specifically, the presence of MPs in beverages was checked through the application of a novel method using a fluorophore named 1-pyrenebutyric acid N-hydroxysuccinimidyl ester (PBN), which was confirmed as a suitable technique for the rapid and accurate detection of different plastic polymers in water samples [23]. The use of staining dyes, including fluorescence-based methods, represents a cost-effective, simple, and easy approach for screening the presence of plastic particles [23].

## 2. Materials and Methods

### 2.1. Isolation of Microplastics (MPs)

A total of 14 nonalcoholic beverage samples covering 14 different brands of soft drink and cold tea were purchased in a supermarket in Northern Italy. All the beverages were liquid. Three samples were cold tea, while 11 samples were soft drinks (i.e., cola, soda, tonic water, and other typologies of soft drinks). All the samples were packaged in transparent plastic bottles made of polyethylene terephthalate (PET) with a polyethylene (PE) cap of a different color. Three bottles of each beverage were purchased to measure MP contamination in triplicate. Beverage samples were processed for MP isolation according to the method described by [24]. To prevent external MPs contamination, all the glassware equipment (i.e., beakers, funnels, and filtering units) and stainless-steel forceps and pins used during the analytical procedure of MPs isolation were preliminary washed with acetone and ultrapure water filtered on cellulose filters (StonyLab, Ø = 47 mm, pore size 0.45 µm) and then wrapped in tinfoil prior to use. All the solutions (i.e., ultrapure water, sodium chloride, and hydrogen peroxide solutions) used for the procedure of MP isolation were preliminary filtered on cellulose filters (StonyLab, Ø = 47 mm, pore size 0.45 µm). Operators wore white laboratory coats made of cotton fabric.

After a gentle shaking of the bottle, 500 mL of each beverage was transferred to a 1 L beaker, and 180 g of sodium chloride was added to saturate the solution (density of the solution = 1.2 g/cm^3^). No dilution of beverage samples was performed. Sodium chloride was solubilized at 90 °C for 30 min through constant stirring with a plastic-free magnetic stirring rod. The solution was then transferred to a 1 L glass separation funnel. The beaker used to perform flotation of MPs through the NaCl-saturated solution was rinsed three times with ca. 20 mL of a NaCl solution in order to collect any residual items. The washing aliquots were transferred to the same separation funnel containing the sample, and the whole solution was allowed to settle overnight. After removing the solution at the bottom of the funnel, the upper phase (ca. 100 mL) containing floating items was added with 100 mL of a filtered 30% hydrogen peroxide solution to reduce the load of colorants and organic matter. This process lasted overnight. The solution was then filtered on cellulose filters (StonyLab, Ø = 47 mm, pore size 0.45 µm) through a filtration apparatus using a vacuum pump operating at pressures of ca. 0.5 bar. The separation funnel was rinsed three times with 20 mL of filtered ultrapure water, and these aliquots were filtered on the same filter used for the sample. Whenever the filters got clogged or blocked, a new filter was used for the filtration of the remaining volume of the solution. Following filtration, the filters were carefully transferred to a petri dish (Ø = 50 mm) using stainless-steel forceps and dried at room temperature for 24 h prior to the isolation of putative MPs.

### 2.2. Quality Control and Assurance

Ten samples of beverages and a blank (i.e., a batch) were processed contemporarily. One liter of filtered ultrapure water was processed according to the procedure used for beverage samples as a procedural blank. All the samples, including blanks, were processed in triplicate. In the blank samples, 9 ± 4.24 fibers were detected, but they were then identified as non-plastic items. 

### 2.3. Identification of Microplastics (MPs)

**Figure 1:**
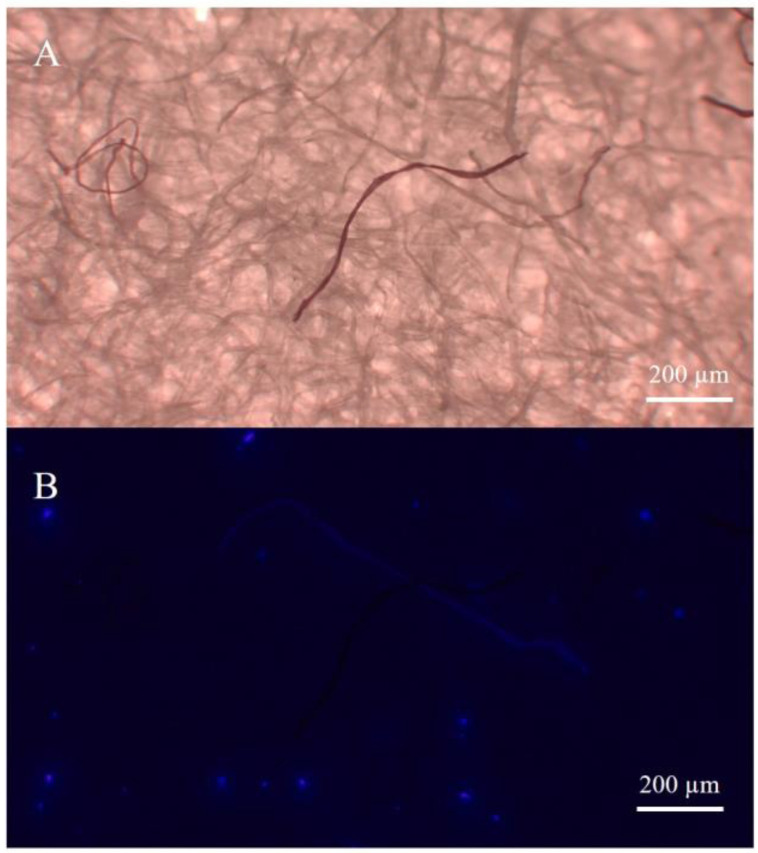
Microscopy images of a filter with fibers isolated from a soft drink before (**A**) and after (**B**) labeling with PBN. Only the plastic fiber emitted electric blue fluorescence.

A visual inspection of the filters from all the beverage samples was performed under a Leica EZ4W stereomicroscope according to the shape and color of the filtered items to isolate putative MPs. Each item that was identified as a putative MP was transferred to a new cellulose filter, i.e., a filter for each replicate of each beverage (including blanks), with stainless steel pins. A picture of all the filters was captured at different magnifications (8× and 16×) and processed through the ImageJ freeware software (version 1.52p, http://imagej.nih.gov/ij/, accessed on 20 January 2023) to measure the length of each putative MP according to the longest dimension. Putative MPs were categorized as fibers or fragments and grouped according to their color. Although the most studies aimed at confirming the polymeric composition of MPs used analytical techniques such as Fourier-transform infrared spectroscopy (FTIR), Raman spectroscopy or Gas Chromatography-Mass Spectrometry techniques [25], to identify the actual from the putative MPs, the identification of the polymeric composition of isolated items was performed through the application of the fluorophore, the 1-pyrenebutyric acid N-hydroxysuccinimidyl ester (PBN). A recent study has confirmed the PBN as a rapid, simple, cost-effective, and highly efficient detection method for the identification of different plastic polymers, such as PE, PET, polyamide-6 (PA-6), polypropylene (PP), polystyrene (PS), polyvinyl chloride (PVC), polycarbonate (PC), polyvinylidenechloride (PVDC), and polyurethane (PU) in bottled water and environmental freshwater samples [23]. In addition, our preliminary analysis confirmed that the PBN did not label items made of natural-based polymers (e.g., cellulose-based fibers). Considering that previous studies demonstrated that the majority of microplastics detected in beverages were fibers made of polyester [10,22], to prove the reliability of the identification approach, we labeled cotton- or polyester-based fibers with the PBN. Fibers of both polymers were collected through the laundering of five black t-shirts made of cotton or polyester (PL) fabrics in a Candy Smart CTDF 1006 6-Kg A+ Energy domestic washing machine, using the washing program specific for synthetic clothing (1:19 h at 30 °C and a final centrifugation at 800 rpm). To allow the collection of fibers, t-shirts were wrapped in a washing bag (i.e., a GUPPYFRIEND^®^ washing bag), which prevented the loss of microfibers from clothes during laundering. At the end of the washing, fibers released from t-shirts that remained in the washing bag were collected and used for our experiment. Ten polyester and ten cotton fibers were transferred to two separate cellulose filters and labeled with PBN. The PBN solution was prepared in dimethyl sulfoxide (DMSO; 100 μg/mL) and filtered using a polytetrafluoroethylene syringe filter (PTFE, 0.22 μm, Whatman) prior to use. All the fibers were labeled, drop-by-drop with 100 μL of the PBN solution, and incubated for 5 min in the dark. After the incubation, the fluorescent labeling of each item was assessed through fluorescent microscopy using a Leica DM4500B microscope coupled with a fluorescence module Pred DM4 (Leica Microsystems Ltd., Wetzlar, Germany). The samples were observed under a DAPI (4,6-diamidino-2-phenylindole) filter at excitation and emission wavelengths of λ = 359 nm and λ = 457 nm, respectively. PBN did not label cellulose-based fibers, while polyester fibers emitted blue fluorescence (Figure 1). Then, five polyester and five cotton fibers were transferred to the same cellulose filter and incubated with PBN as above. As expected, only polyester fibers were labeled and emitted fluorescence.

### 2.4. Statistical Analysis

As the Kolmogorov-Smirnov test revealed that the data did not follow a normal distribution, the differences in the number of MPs among soft drink and cold tea beverages, as well as among different types of beverages grouped in five categories (i.e., cola, soda, tonic water, cold tea, and others), were assessed through the application of the non-parametric Kruskal-Wallis test. Statistical analysis was run in R 3.6.1 [26].

## 3. Results

**Figure 2:**
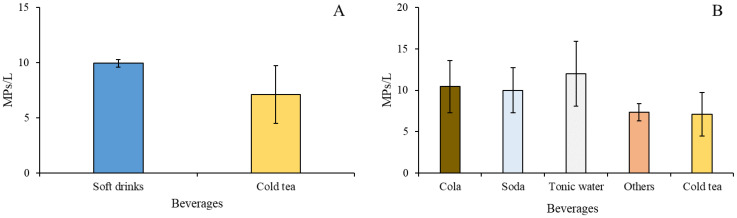
Mean concentration of MPs (±SEM) was measured in soft drink and cold tea samples (**A**) and in different groups of beverages (**B**). Three different brands of beverages per group were tested.

The results of the present study confirmed the presence of MPs in nonalcoholic beverages, both in soft drinks and cold tea. Our preliminary visual inspection isolated items attributable to MPs (hereafter “putative MPs”) in all the analyzed samples, for a total of 394 putative MPs. In detail, 345 putative MPs were isolated from soft drinks (range: 1–27 items) and 45 from cold tea (range: 1–18 items) samples. The mean size of putative MPs was 495 µm (range: 36–2228 µm); most of them (91%) were fibers, while only 9% were fragments. Black was the dominant color among putative MPs (53%), followed by blue (15%) and transparent (9%). After the application of PBN fluorophore, the number of items made of plastic polymers accounted for 189 items, corresponding to 48% of putative MPs. Among them, 157 were found in soft drinks (ranging from 0–15 items) and 32 in cold tea (ranging from 1–10 items). Only 5% of the MPs were fragments (n = 11), while 95% were fibers (n = 178). The mean (± standard error of the mean; SEM) number on MPs (expressed as MPs/L of beverage), independently of the type of beverage, was 9.19 ± 1.84 MPs/L, while specifically for soft drinks and cold tea was 9.94 ± 0.33 MPs/L and 7.11 ± 2.62 MPs/L, respectively. There was no significant differences in the amount of MPs (expressed as MPs/L) between soft drinks and cold tea (H = 1.429, degrees of freedom = 1, N = 42; *p*-value = 0.232) (Figure 2A). Similarly, no differences in the amount of MPs occurred among the different types of beverages grouped into five categories (i.e., cola, soda, tonic water, cold tea, and others) (H = 1.976, degrees of freedom = 4, N = 42; *p*-value = 0.740; Figure 2B).

## 4. Discussion

A growing number of studies has confirmed the ubiquitous presence of MPs of different size, shape, color, and polymeric composition in the environment as well as in the food we eat. The polymers dominating the composition of MPs isolated from food samples were PET, PE, PP, and PS [27], which are the main polymers used in food packaging and correspond to the largest amount of plastic demand worldwide [28]. Similarly, some studies have revealed the presence of MPs in drinking water [16] and beverages, including dairy milk [22], beer [21], soft drinks [10], and wine [29]. Our results confirmed that nonalcoholic beverages, namely soft drinks and cold tea, were contaminated by MPs. On average, 9.19 ± 1.84 MPs/L (range: 0–30 MPs/L) were detected independently of the beverage type. Microplastics were detected in all the brands and types of beverages except for cola samples of a single brand, which were not contaminated. Our results confirmed diffuse contamination by MPs in beverages from the Italian market, and they were consistent with those reported in a previous study performed in Mexico, where the amount of MPs observed in soft drinks and cold tea purchased in markets was in the 0–7 MPs/L and 1–6 MPs/L ranges, respectively [10]. However, the contamination levels observed in both the studies mentioned above were lower compared to those reported in an investigation of MPs in soft drinks purchased in markets from Ecuador (32 MPs/L) [30]. All the studies on beverages, independently of the country in which they were bought, confirmed that the pattern of contamination was dominated by fibers, while the contribution of fragments was very limited [10,21,22,31].

Different factors related to operational and production processes can contribute to MP contamination in beverages. First, contamination of beverages by MPs can originate from the water used in the production process. It has been estimated that about 3–4 L of water are necessary to produce 1 L of soft drinks [32], and a greater amount of water is used to produce and/or clean containers, for washdown operations at production plants, and for the washing of bottles [32]. Thus, the presence of MPs in freshwater, tap, and drinking water [16,33] used during productive processes, coupled with the overall contamination of working areas due to the presence of airborne items, might contribute to beverage contamination [10]. Other contributors can be identified in the packaging materials of bottles and bottle caps. For instance, breakage and degradation over time of the plastic polymers used to manufacture beverages and water bottles, the stress applied to the bottles, as well as the opening/closing of bottle caps, can result in the release of MPs [17,34,35]. Additional sources of MPs can be identified in membrane filters used in the industrial production of foods and beverages [22]. Lastly, another important contribution to MP contamination can result from the atmosphere, which is considered a vector of global MP dispersion but is also a potential source of exposure for organisms and humans through breathing, ingestion [36], and food contamination [37]. MPs were detected in outdoor and indoor atmospheres [9,38], whereby fibers of different polymeric compositions, including PL, PET, PA, PE, PP, and PS, coming from the outdoor environment or wearing clothes and materials used in production plants dominated the contamination pattern [39]. Overall, the concentrations of MPs detected indoors were generally higher compared to outdoor ecosystems due to the different sources of contamination and mechanisms involved in their dispersion, such as ventilation, air flow, and climatic conditions [38]. Thus, MP deposition to the ground can contaminate working surfaces and also products during the different production steps, resulting in beverage contamination. To date, no study has monitored indoor contamination of MPs in beverage factories. However, previous surveys showed that the fingerprint of MP contamination observed in beverages was similar to that characterizing the indoor air, which was dominated by fibers made of PL, PET, and PA [10,29,39].

The presence of MPs in beverages, as well as in drinking water and food, can result in their continuous intake by humans [40]. Although human health effects induced by the ingestion of MPs are unlikely, long-term consequences are unknown [41]. Hence, to estimate the intake of MPs due to beverage consumption, the ingestion of MPs on a daily and yearly basis was estimated. As the 14% of young people in the 15 to 24 age group declared a daily consumption of soft drinks, we performed a brief survey to assess the amount (i.e., number of glasses and weekly frequency of consumption) of soft drinks consumed in a sample of students (aged 17–21; n = 42). On average, two glasses of soft drinks (ranging from 0–6 glasses) were consumed two days a week (range: 0–7 days a week). Considering that a glass of water holds approximately 200 mL of volume, according to our results on MPs contamination in beverages, the mean estimated amount of ingested MPs was 7.49 MPs/week (ranging from 0–252 MPs/week depending on the number of glasses and the frequency of consumption). Focusing on soft drinks only, the mean estimated amount of ingested MPs was 8.10 MPs/week (range: 0–252 MPs/week), and assuming a daily consumption of soft drinks, the annual ingestion of MPs should amount to 421.67 MPs (range: 0–13,104 MPs). Our estimates were lower than those reported by a previous study that performed a rough approximation of daily and annual MPs consumption via food and beverages in a sample of American young and adults, with daily and annual MPs consumption ranging from 106 to 142 and 39,000 to 52,000, respectively [40]. Although these estimates were likely underestimated [40], they were higher than those we calculated because they also included MPs ingestion from food and were not exclusively related to beverages. Another factor of uncertainty in the estimates is the large inter-individual variability in MP size, number, and frequency of ingestion, which can be influenced by dietary habits, food types, countries, or regions [42]. According to these considerations, diverse studies agreed on demonstrating a huge variability in the number and composition of MPs ingested by humans. Although a recent investigation confirmed that ingested (or inhaled) MPs can be eliminated through the feces [8], there is a dearth of information about their fate and permanence within the human body. For these reasons, as well as considering the potential presence of additives included in or absorbed by MPs, potential consequences for human health due to MP ingestion cannot be excluded but, at present, are completely unknown and require further investigations.

## 5. Conclusions

Our results confirmed the presence of MPs in nonalcoholic beverages, whose consumption can be considered one of the main routes for their ingestion by humans, specifically by young people, who are the main consumers of these products. In addition, our study suggests that staining and/or fluorescence-based methods can be considered as rapid, cheap, and sensitive approaches for screening and identifying the presence of plastic items, including MPs, in beverages and water samples. MPs were detected in the vast majority of beverages we processed, suggesting a ubiquitous contamination that can originate from different sources, including water used in beverage production or washing procedures in the plants, contamination of the working area, and airborne contamination. Considering the different sources of contamination, the presence of MPs in beverages might push the producers and the industry to identify the sources and prevent the contamination in these products, as well as implement elimination measures. On one hand, these strategies should allow for the prevention of contamination of beverages and ingestion by humans due to beverage consumption; on the other hand, they should reduce airborne contamination and MPs intake through inhalation. Although the human intake of MPs widely differs among individuals depending on dietary habits, food types, or regions, the reduction of MP contamination in beverages and the workplace should prevent potential adverse human health effects caused by exposure to these emerging contaminants. For these reasons, further studies on MPs’ ingestion through food, drinking water, and beverages, as well as their fate and permanence in the human body, should be a priority to estimate the exposure and the potential hazard to human health.

## Data Availability

Data will be shared upon request.
